# A diffusion model for the coordination of DNA replication in *Schizosaccharomyces pombe*

**DOI:** 10.1038/srep18757

**Published:** 2016-01-05

**Authors:** T. Pichugina, T. Sugawara, A. Kaykov, W. Schierding, K. Masuda, J. Uewaki, R. S. Grand, J. R. Allison, R. A. Martienssen, P. Nurse, M. Ueno, J. M. O’Sullivan

**Affiliations:** 1 Liggins institute, University of Auckland, Grafton, Auckland 1032, New Zealand; 2 Research Center for the Mathematics on Chromatin Live Dynamics (RcMcD), Hiroshima University, 1-3-1, Kagamiyama, Higashi-Hiroshima, 739-8530, Japan; 3 The Rockefeller University, New York, New York 10065, USA; 4 Department of Molecular Biotechnology, Graduate School of Advanced Sciences of Matter, Hiroshima University, 1-3-1 Kagamiyama, Higashi-Hiroshima, 739-8530, Japan; 5 Centre for Theoretical Chemistry and Physics, Institute of Natural and Mathematical Sciences, Massey University, Albany, Auckland 0632, New Zealand; 6 HHMI-GBMF, Watson School of Biological Sciences, Cold Spring Harbor Laboratory, 1 Bungtown Road, Cold Spring Harbor, New York 11724, USA; 7 The Francis Crick Institute, Lincoln’s Inn Fields Laboratories, London WC2A 3LY, United Kingdom; 8 Gravida: National Centre for Growth and Development, University of Auckland, Auckland, New Zealand

## Abstract

The locations of proteins and epigenetic marks on the chromosomal DNA sequence are believed to demarcate the eukaryotic genome into distinct structural and functional domains that contribute to gene regulation and genome organization. However, how these proteins and epigenetic marks are organized in three dimensions remains unknown. Recent advances in proximity-ligation methodologies and high resolution microscopy have begun to expand our understanding of these spatial relationships. Here we use polymer models to examine the spatial organization of epigenetic marks, euchromatin and heterochromatin, and origins of replication within the *Schizosaccharomyces pombe* genome. These models incorporate data from microscopy and proximity-ligation experiments that inform on the positions of certain elements and contacts within and between chromosomes. Our results show a striking degree of compartmentalization of epigenetic and genomic features and lead to the proposal of a diffusion based mechanism, centred on the spindle pole body, for the coordination of DNA replication in *S. pombe*.

The *Schizosaccharomyces pombe* genome contains a range of histone modifications associated with active (e.g. H3K4me) and inactive genes (e.g. H3K9me)^1^,^2^,^3^,^4^. Histone H3 methylated at Lys9 (H3K9me) defines heterochromatin and is enriched at sequences surrounding the centromeres, subtelomeres, ribosomal DNA (rDNA) and mating type locus^2^. By contrast, histone H3 methylated at Lys4 (H3K4me) defines euchromatin and is distributed across the *S. pombe* genome while being absent from the major heterochromatic loci that are covered by the H3K9me mark^2^. Despite this, it remains unclear if these modifications are spatially segregated within the nucleus.

DNA replication, like transcription, is widely recognized as occurring at punctate sites within the nuclear space^5^,^6^. Origins of replication in *S. pombe* are similar to those in higher eukaryotes in that they do not share any identifiable consensus elements^3^. Yet, *S. pombe* origins of replication can be classified according to their times and efficieny of firing^3^,^5^. Specifically, origins in the pericentromeric heterochromatin fire early while those in the telomeric, mat locus and rDNA regions replicate later than euchromatic origins^3^,^7^. However, at the level of the linear chromosomal sequence, the selection of the origins of replication that fire is stochastic^5^,^8^. Therefore, it remains likely that spatial information is required to understand the mechanisms that result in the seemingly inefficient and non-deterministic selection of eukaryotic origins of replication.

While proximity ligation methods have revealed colocalisation of unlinked loci within a genome, three-dimensional (3D) models are required to interpret this data in terms of a description of the spatial organization of the epigenome. Our approach is to use coarse-grained polymer models of the chromosomes that are integrated with empirical measures of genome organization (Fig. 1). We generated a population of 1000 independent 3D structures of the *S. pombe* genome using a coarse-grained polymer model that incorporates: the chromosome flexibility; the locations of centromeres and telomeres within the nucleus; and the chromosomal contacts captured by proximity-ligation. The chromosomal contacts were captured in *S. pombe* cells that were synchronized in the G1 and G2 cell cycle phases^9^,^10^, unsynchronized wild-type cells, and unsynchronized cells lacking the Clr4 methyltransferase (Δ*clr4*)^10^. The procedure used to generate these structures accounts for the probabilistic nature of the proximity-ligation methods that were used to capture the chromosomal contacts (*methods*). A detailed explanation of the choice of paramters for these models is provided in^9^. The modeled structures were validated by comparing the distributions of distances between the spindle pole body (SPB) and five loci (*i.e. Ade3, His2, Ade6, Lys1*, and *Ade8*) calculated from our synchronized G1 and G2 phase models with *in vivo* microscopic measurements (Figs S1 and S2). While it should be recognized that our models have a number of parameters that may yet be further optimized, the overlap between the distributions was in general comparable and better for the G2 phase data (compare Figs S1 and S2 *Ade8*). The observed deviations may be explained by a combination of modelling parameters and the fact that the microscopic measurements were made on live unsynchronized cells, bearing in mind that *S. pombe* only has a short G1 phase and spends ~70% of the time in the G2 phase of the cell cycle. However, the general agreement we observed encouraged us to interrogate the structures further.

We determined the spatial positioning of dimethylated histone H3 Lysine 4 (H3K4me2) (euchromatin) and histone H3 Lysine 9 (H3K9me2) (heterochromatin) within the *S. pombe* nucleus (Fig. 1A–C,E). Linear positions of the H3K4me2 and H3K9me2 epigenetic marks were determined by ChIP-chip (chromatin immunoprecipitation microarrays)^2^ and projected onto our ensembles of *S. pombe* genome structures. The localization density of H3K4me2 or H3K9me2 reveals spatial separation of euchromatin and heterochromatin (Fig. 1C,E). Heterochromatic loci are enriched at the nuclear periphery while euchromatin is enriched towards the center of the nucleus. Incorporation of biological restraints in the modeling procedure significantly alters the spatial distributions of these loci, indicating that nuclear confinement (*i.e.* random model, Fig. 1C,E) is insufficient for euchromatin and heterochromatin segregation and emphasizing the importance of including the chromosome contact data in the modeling procedure. The growth medium only had minor effects on these spatial distributions (Fig. S3). Interestingly, we observed a degree of spatial overlap of euchromatic and heterochromatic loci within our models, in that co-enrichment of both euchromatic and heterochromatic marks was observed in the vicinity of the spindle pole body (SPB) and coincided with centromere proximal chromosome sequences (Fig. 1C,E, Fig. S4). The significance of this spatial overlap remains to be determined. It is possible that the spatial overalp in the vicinity of the SPB reflects: 1) the resolution (*i.e.* granule size) of the models; 2) the projection of the 3D *S. pombe* nucleus onto 2D maps may cause regions that were separated by rotation in space to appear to be close together; or 3) the loss of temporal information that separates when each modification occurs. However, it is also possible that the spatial overlap in the vicinity of the SPB represents a novel combinatorial aspect of epigenetic control that requires the simultaneous or sequential consecutive presence of these marks that were previously considered mutually exclusive^2^.

Argonaute (Ago1), chromodomain protein (Chp1), RNA-dependent RNA polymerase (Rdp1), and the HP1 heterochromatin protein homologue (Swi6) contribute to the formation and maintenance of *S. pombe* heterochromatin (reviewed in^11^,^12^). Our models show that loci bound by these proteins colocalize in the heterochromatic perinuclear region (Fig. 1D and Fig. S5; >90% coincidence). The population level distribution of loci bound by Swi6 is consistent with microscopic observations that show foci in a peripheral location^13^, albeit no one cell has Swi6 foci spread across the entire enriched region. Crucially, the observed overlap between the Ago1, Chp1, Rdp1 and Swi6 protein binding sites and the heterochromatic (H3K9me2) loci increased in the spatial models when compared to the overlap that was observed in the linear sequence (Fig. S5). Moreover, this spatial overlap was specific for H3K9me2-modified loci (Fig. S5). By contrast, the CENP-B homologues, Abp1 and Cbh1, which bind and silence retrotransposons^1^, did not show increased spatial colocalization in our models (Fig. S6). The observed spatial positioning, segregation and co-localization of the different epigenomic features in our contact-restrained model genome structures demonstrates that models of 3D genome structure that are constrained by DNA flexibility and centromere, telomere, and nucleolar positioning, coupled with experimentally-derived chromosomal contact data, can recreate biological spatial positioning.

Next we considered the spatial distribution of replication origins classified according to their time of firing in the S phase of the cell cycle and their frequency of use (*i.e.* efficiency) within a population of cells^3^,^14^. There was clear spatial segregation of origins of replication according to their firing times (Fig. 2A); early-firing origins overlap regions enriched for euchromatic loci, except for a cluster at the SPB that is consistent with the early S phase firing of pericentromeric origins of replication (compare Figs 2A and 1E). By contrast, late-firing origins are located towards the nuclear periphery and nucleolar boundary in regions that are enriched for heterochromatic loci (Figs 2A and 1C,D). The prominent spatial colocalization of Abp1 and Cbh1 with the euchromatin (Fig. S7) and early-firing origins of replication is consistent with their role in retrotransposon silencing, co-localization^1^,^2^, replication^15^ and replication fork restart^16^. Origins of replication also colocalized in groups according to their firing efficiencies, with high efficiency origins being enriched in regions around the nuclear center and SPB (Fig. 2B). By contrast, medium efficiency origins, which fire once every five cell cycles, are preferentially located towards the nucleolus along the SPB-nucleolar axis (Fig. 2B). Finally, low efficiency origins, which fire once every 10–20 cell cycles, are located towards the nuclear periphery in regions enriched for heterochromatic loci (Figs 2B and 1D). Once again there is clear overlap of high, low and medium efficiency origins in the vicinity of the SPB. Collectively, our results are consistent with the existence of discrete heterochromatin environments that affect origin firing and efficiency in a distinct and separable manner^17^,^18^,^19^.

The spatial segregation of euchromatic and heterochromatic loci within the 3D nuclear architecture observed in our models is striking. Clr4 is the sole histone H3K9 methyltransferase in *S. pombe* and is considered to be essential for heterochromatin formation^2^. Therefore, by comparing the positions of loci that were shown to be heterochromatic and euchromatic in wild-type (WT) *S. pombe* cells between the Δ*clr4* and WT genome models, we were able to monitor the effect of a loss of H3K9me2 on global genome structure. Surprisingly, in the Δ*clr4* models there were only minor differences in the spatial distribution of loci that were decorated with heterochromatic and euchromatic histone marks in WT cells (Fig. 3A). However, there were considerable alterations to the spatial positioning of the mating-type (mat) locus (Fig. S8) and origins of replication (Fig. 3B–F) consistent with earlier observations of a loss of mat positioning in the absence of Clr4^20^. Crucially, while the positional shifts for the mat locus appear random, there was a clear reciprocity in the shifts of the early- and late-firing origins (Fig. 3B) and the high and low efficiency (Fig. 3C) origins of replication, consistent with defects in the firing of early origins of replication in Clr4 mutants^16^. However, it was clear that some origins of replication contributed more than others to the observed pattern of changes in density (Fig. 3E,F, Tables S2 and 3). For example, not all late firing origins move towards the nuclear center following deletion of Clr4, consistent with our finding that global structure is not significantly affected by deletion of Clr4 (Fig. 3D). High efficiency origins of replication on chromosome 3 and surrounding the centromeres of chromosomes 1 and 2 tended to move out from the nuclear interior following deletion of Clr4 (Fig. 3E). Low efficiency origins of replication that migrated into the nuclear interior tended to have more telomeric positions on the chromosomes (Fig. 3F). The observed changes to the spatial positions of the mat locus and replication origins reflect the stable deletion of Clr4 and not the loss of cell synchronization (Figs S8 and S9).

Kaykov and Nurse previously showed that the individual and combined deletion of Clr4 and Clr3 (histone deacetylase) disrupts the nuclear organization of replication in *S. pombe*, with the deletion of Clr4 exhibiting the stronger effect^5^. We reasoned that the reciprocal movement of high efficiency and low efficiency origins of replication predicted by our ∆*clr4* models (Fig. 3C) would correspond to a change in their efficiency. Therefore, we measured the genome wide reprogramming of replication origin usage in ∆*clr3*∆*clr4* cells (Fig. 4). We observed a global decrease in the efficiency of origin replication (Fig. 4). This was accompanied by a decrease in the relative efficiency of efficient origins (Fig. 4C) and an increase in the relative efficiency of inefficient origins (Fig. 4D). These results are consistent with the spatial positioning of origins and chromatin structure within the nucleus having a role in global replication timing^5^. Such a role might be mediated through interactions between Clr4 and the RNA polymerase II machinery acting to coordinate replication and transcription^16^, in addition to Clr4’s role in replication timing at pericentromeric regions^21^. Moreover, the origin specific effects observed in the *∆clr4* strain are consistent with replication timing not simply being linked to histone methylation^21^. Instead, our results are consistent with the global structuring of the pericentromeric and telomeric heterochromatin occurring independently of H3K9me2, but that positioning of specific loci and origins of replication is dependent upon the histone H3K9 methyltransferase.

Models that purport to explain the global organization of origin firing must cope with the apparent lack of a deterministic program for origin of replication firing, specifically: a) origins do not fire every cycle; b) there is no linear clustering of active origins; and c) there is no obvious epigenetic programming of origin firing to increase the likelihood of any one origin firing in subsequent cycles^5^,^8^. The spatial organization of origins of replication within our models of nuclear structure in the G1 phase of the cell cycle, and in particular the apparent delay in timig and reduction in efficiency according to the distance from the SPB, lead us to hypothesize that chromatin organization in conjunction with a SPB-originating diffusion mechanism coordinate origin firing (Fig. 5), consistent with observations from earlier studies^5^. In our hypothesis the G1-specific chromatin organization locates the positions of replication origins so that a mobile activation factor(s), which is modified near the SPB, can diffuse out to search the nuclear architecture for replication origins before activating them. Numerous potentially diffusible limiting factors for replication have been described (e.g.^22^). In this model, selection of an origin for activation is random, but the timing and efficiency are determined by: 1) proximity of the origin to the SPB determined by the 3D genome organization; and 2) local chromatin architecture affecting the speed of diffusion and ability of the mobile activation factors to explore the DNA^23^. Thus, the relative stability of the genomic structure through the G1-S-G2 phases of the cell cycle^9^ – excluding remodeling in response to environmental perturbations – means that our model incorporates random selection of origins while maintaining the early/late firing split and differential efficiencies by incorporating dependance on the proximity of the origin to the SPB. The fractal nature of the folded nuclear chromosomes prevents the formation of gaps within the replicated structure by promoting the selection of origins from across the linear sequence of the chromosomes. Subsequently, the initial selection of the origins seeds the activation of origins in both spatial and linear proximity – to create replication foci, consistent with earlier observations^5^. The role of the SPB (or centrosome) in our model is consistent with their widely recognized role as signalling centres^24^ and suggests a role in coordinating passage through the interphase of the cell cycle.

## Methods

### Chromosome contact data

Chromosome contact data was obtained from^9^. Briefly *S. pombe* MY291 (h- lue1 cdc10-129) cells were synchronized by temperature shift and the chromosomal contacts captured using genome conformation capture (GCC)^9^,^14^. GCC networks were constructed from 50 bp paired-end Illumina Genome Analyser sequence reads using the Topography suite v1.19^9^.

### Chromosome features, epigenetic and protein binding sites

Published ChIP-chip studies were mined to obtain ChIP enrichment data for H3K9me2, Ago1, Swi6, Chp1, Rdp1, H3K4me2^2^, Abp1, and Cbh1^1^. The chromosomal locations, firing times, and efficiency of origins of replication were obtained from^3^.

Chromosome coordinates for earlier genome versions were converted to coordinates for the *S. pombe* reference genome (ASM294v2) using LiftOver^25^. To LiftOver coordinates from the various genomes to our reference, chain files were created as described (http://genome.ucsc.edu/admin/git.html and^25^). Briefly, fasta files from each different reference were compared with BLAT to align similar sequences from each chromosome and create a LiftOver chain file. This file then was used as input into LiftOver to change coordinates from the earlier genome reference to the one used for this analysis. 40623 out of 41173 locations could be remapped, with 550 locations of methylation lost due to the change in reference.

### Strain construction

The strains used in this study are listed in Table S1. KM023 was created by mating between FY15592 and FY14816. KM026 was created by mating between FY15591 and FY14816. KM025 was created by transformation of FY15550 with the *sid4::sid4-GFP-HA-Kanr* fragment, which was PCR amplified from strain FY14816 using the following primers: ATGACGGGTCTACAGCCCC and CATGGGCTGAAAAGATATAATG.

### Microscopy

Cells were grown in YEA medium (0.5% yeast extract, 3% glucose, and 40 μg/mL adenine) at 30 °C (o/n). 2.5 × 10^5^ cells, from a log phase culture, were added to a glass-bottom dish (Iwaki; coated with 5 mg/mL lectin from *Bandeiraea simplicifolia* BS-I (Sigma)) for live cell imaging. A stage top incubator with lens heater (HoKai Hit) was used to maintain sample temperature at 30 °C. Each sample was used for a maximum three hours of imaging before cells reach stationary phase.

Microscopic images of asynchronous cells were obtained using an iXon3 897 EMCCD camera (Andor) connected to Yokokawa CSU-W1 spinning-disc scan head (Yokokawa Electric Corporation) and an OlympusIX83 microscope (Olympus) with an UPlanSApo 100× NA 1.4 objective lens (Olympus). Pictures were captured and analyzed using MetaMorph Software (Molecular Devices). Optical section data (41 focal planes with 0.2 μm spacing every 30 sec.) were collected. Time-lapse sequences were deconvolved using Huygens image analysis software (Scientific Volume Imaging).

### Quantitative analyses of the distance between SPB and LacO-labeled genomic loci

Microscope images of the interphase cells were used to examine the distance between SPB and LacO-labeled genomic loci. Deconvolved time lapse sequences for each locus were analyzed using the IMARIS software (Bitplane). The center of the fluorescent focus was tracked over 30 minutes at 30 second intervals for each strain. In order to remove the movement of the nucleus, the distance between SPB and LacO-labeled genomic loci was calculated. The distribution of the distance was obtained using the following number of data points: n = 530 for *lys1,* n = 2877 for *ade6*, n = 3595 for *his2*, n = 2645 for *ade3*, n = 2507 for *ade8*, n = 2249 for *sod2*.

### Coarse-grained polymer modeling

G1 phase *S. pombe* chromosomes were modeled as worm-like polymer chains confined to lie within the nuclear space, excluded from the nucleolus, and subjected to both tethering restraints (*i.e.* centromere and telomere positioning) and inter- and intrachromosomal interaction restraints derived from synchronized *S. pombe* MY291 cells^9^,^10^.

The genome was represented by 3583 granules, each of 30 nm diameter and representing 3.5 kb of DNA. Granules were strung together into three separate polymer chains, representing chromosomes 1, 2 and 3; overlaps were forbidden to reproduce the excluded volume of the chromosomes.

Chromosome configurations were subjected to spatial constraints governing the nuclear size and exclusion from the nucleolus^26^. Biological constraints restricting telomere^27^ and nucleolar positioning, and centromere colocalization with the spindle pole body (SPB)^27^,^28^,^29^ were based on microscopic observations. Random model genome structures incorporated only nuclear confinement and no other biological restraints.

The contact-restrained *S. pombe* genome models also included captured non-adjacent interactions from the GCC^9^ and Hi-C data^10^. GCC and Hi-C captures the chromosomal connections that are occurring within a population of cells^9^,^30^,^31^. As such, it is impossible to determine which chromosomal connections are occurring within the single cell. Therefore, each structure generated for the interactions model used a subset of the captured interactions that was randomly selected with a probability proportional to their detection frequency. Thus, connections that occur more frequently within the population are more likely to be captured by GCC and HI-C , and incorporated into the models as attractive forces^9^. However, every captured interaction was present in at least one model in the ensemble.

To be able to compare between the ensembles of structures generated for the different data sets, the mean radius of gyration of the modeled genome structure was fixed to half the nuclear radius (650 nm). The size of the subset of interactions^9^,^10^ used in the modeling procedure was chosen as the number of interactions that reproduced the chosen radius of gyration. The subset size for interactions obtained in GCC experiments^9^ was equal to 10% of the total interactions set. The subset size for interactions obtained in Hi-C^10^ experiments was in the range (7.7 × 10^−3^%, 1 × 10^−2^%) of the total interactions set. Thus, we overcame differences in the underlying methods that were used to capture the chromosomal contacts^9^,^10^.

Chromosomes structures for the random and interactions models were optimized using a Monte-Carlo approach^9^. An ensemble of 1000 independently optimized genome structures was generated for each model. Each ensemble of structures represents a spectrum of possible genome configurations within the cell population.

### Translation of experimental data onto the models

The positions of genetic elements and ChIP-chip enrichment sites were converted from linear sequence coordinates into granule positions. Since each granule represents 3.5 kb of sequence, several genetic elements or enrichment sites could be incorporated into a single granule. A binary system was used to map the positions of genetic elements (*e.g.* origins of replication) onto granules. Thus, if the genetic element of interest were present in the granule it was given a signal value of 1, while granules that do not contain the element of interest were designated 0.

The inclusion of low enrichment sites from the ChIP-seq and ChiP-chip data may hide patterns of preferential associations for histone or protein enrichments within the nuclear space. This reflects a central challenge in the analysis of ChIP-seq and ChIP-ChIP data: how are biologically meaningful sites differentiated from statistically significant sites?^32^ Moreover, the resolution of our model is limited to 3583 granules, each representing 3500 bp of DNA. Thus including all the identified binding sites for any one modification results in a loss of ability to discriminate discrete zones of enrichment, as too many granules become labelled. Therefore, we limited our analysis to the top 5% of ChIP signals which were extracted from http://pombe.nci.nih.gov/genome/rawdata.html and selected for mapping onto the granules. The enrichment value assigned to each granule was calculated as the number of ChIP-chip peaks within the granule. This approach was adopted for the datasets where the ChIP-chip probes were 300 bp in length^1^,^2^. In this instance, the number of peaks within the granule represents the size of the enriched region within the granule.

### Relative density maps

Relative density maps were calculated to assess the preferred positions of different elements across the nucleus^9^. These maps show the number of times that a granule to which that element has been mapped occurs at the given point within the nucleus, averaged across an ensemble of structures.

Since G1 phase *S. pombe* nuclei can display rotational symmetry with respect to the SPB-nucleolus axis, the 3D structure was rotationally projected onto a 2D map to facilitate visualization^9^,^33^,^34^. Once mapped onto the 2D plane, we calculated the density of these points across the nuclear space using a 266 × 266 pixel rectangular grid where:


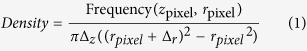



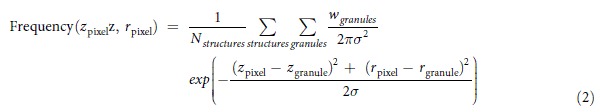


Here ∆_z and ∆_r are the grid size in the z and r directions respectively. *z*_*granule*_ is the projection of the granule onto the SPB-nucleolus axis; and *r*_*granule*_ is the radial distance between the granule and axis of symmetry; *z*_*pixel*_ and *r*_*pixel*_ are pixel grid coordinates; *w*_*granule*_ is the binary signal value for the element of interest: *N*_*structures*_ is the number of structures in the ensemble. *σ* was set to the 15 nm (*i.e.* the radius of granules).

To exclude the effect of general genome compaction due to the model restraints, the density of granules containing the element of interest was normalized by total chromosome density:


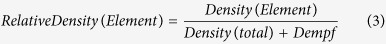


Here *Density (total)* is the ensemble-averaged density of all granules in that pixel and *Dempf* is a small adjustment, which negates a discrete noise in the zones of low absolute density, *e.g.* the nuclear periphery. The value of *Dempf* was chosen as the density of one granule at the nuclear periphery


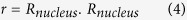


was set to 1.33^9^. In the density maps obtained in our study the majority of the nuclear area has an absolute density 5 times higher than *Dempf*, so the influence of *Dempf* on the relative density can be neglected for most pixels.

### Contour plots

To mark the localization pattern of the element of interest, contour plots were calculated based on the relative density maps. Contour levels were plotted as a curve such that the area within the curve represents the top 15% of signal in the relative density maps. The main advantage of contour plots is that they can be overlaid for different elements of interest.

### Relative density difference plots

To mark the changes in relative densities, the density difference plots were calculated as difference between two particular relative density plots.

### Calculation of the impact of individual chromosome granules on the relative density difference plot

To assess the impact of individual chromosome granules, the relative density plot within the area of interest (i.e. within 670 nm of the SPB-nucleolus axis of rotation) was calculated for each chromosome granule separately. The coordinates of each granule were isolated across the population of the model structures and the relative density maps were calculated, as described above for two selected model ensembles (unsynchronized WT and ∆*clr4*). We calculated the difference for each granule between the corresponding relative density maps derived for the two selected model ensembles. The granules that corresponded to the elements of interest were identified and annotated.

Within the selected region, we identified the pixels that increased or decreased in density, depending on the element we were investigating (*i.e.* high efficiency origins – pixels that decreased were chosen). We then calculated the mean value of the density difference plot for each granule at these pixel positions. The mean % density change for each granule was plotted against the granule position within the *S. pombe* genome. The positions of the elements of interest were marked on these plots.

### Venn diagram illustrating the degree of linear overlap between elements of interest

Venn diagrams were used to visualize the congruence of linear localization patterns on chromosomes between pairs of elements of interest. Each element from the pair is represented by a circle in the Venn diagram, with an area proportional to the number of granules containing the particular element of interest. The area of the intersection between the circles is proportional to the number of granules containing both elements of interest.

### Venn diagram illustrating the degree of spatial overlap

The congruence of the spatial localization patterns on chromosomes between pair of elements of interest was displayed as Venn diagrams. Each element from the pair is represented as a circle on the Venn diagram where the area of the circle is proportional to the area within the contour plot of the corresponding element of interest. The intersection of the Venn diagrams is proportional to the overlap of the contours for the elements of interest. Since the contour plots were limited to 15% of the signal of the relative density maps, the sizes of the two circles comprising the Venn diagrams are equal to each other.

### Micro-arrays

BrdU labeling, immunoprecipitation, array hybridization and data analysis were performed as described^22^. Briefly, *cdc25-22* cultures (PN10499 & PN10607; Table S1) were synchronized at 36.5 °C and released to 25 °C in the presence of 300 μM BrdU (Sigma) and 12 mM hydroxyurea (Sigma), which restricts DNA replication to regions around the origins. Genomic DNA was prepared from unlabeled cells blocked in G2 and from cells harvested at 120 minutes after release. Labeled S-phase DNA and control G2 DNA were sonicated and immunoprecipitated with mouse anti-BrdU antibody (BD Biosciences). Immunoprecipitated DNA was amplified using ligation mediated PCR, biotin labeled with the BioArray Terminal Labeling Kit (Enzo Life Sciences) and hybridized to Affymetrix *S. pombe* Tiling 1.0FR Arrays at 20 base pair probe resolution. The relative enrichment was calculated as the signal ratios of labeled to unlabeled samples and was plotted as a function of the chromosomal coordinates. Two independent sets of experiments were performed.

### Quantitative real-time PCR

Genomic DNA was prepared from *cdc25-22* cells blocked in G2 and from cells arrested in S-phase by adding hydroxyurea (Sigma) at release and harvesting cells at 120 minutes after release. DNA was mixed with SYBR Green PCR mix (Applied Biosystems) and processed with a Roche Lightcycler 480. The CP values for each PCR product were normalized by subtracting the CP value obtained after amplification of one unreplicated region at the conditions used. The relative enrichment in copy number was derived by dividing the normalized CP values obtained from S-phase DNA by the normalized CP values obtained from G2 DNA. The data shown represent the average of two independent biological repeats. The sequences of PCR primers and the chromosomal location of amplified loci are listed in Table S4.

## Additional Information

**How to cite this article**: Pichugina, T. *et al.* A diffusion model for the coordination of DNA replication in *Schizosaccharomyces pombe. Sci. Rep.* **6**, 18757; doi: 10.1038/srep18757 (2016).

## Supplementary Material

Supplementary Information

## Figures and Tables

**Figure 1:**
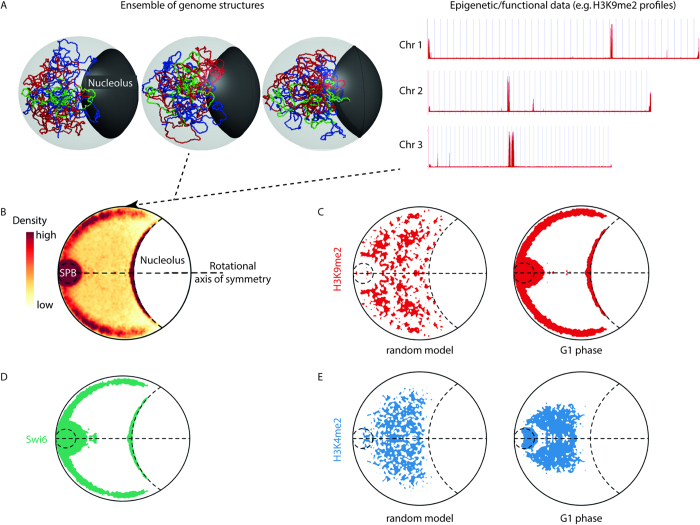
Euchromatin and heterochromatin are compartmentalized within the G1 phase *S. pombe* interphase nucleus. (**A)** Ensembles of 1000 genome structures were generated by polymer modeling^9^. Chromosomal coordinates were identified in ChIP-chip datasets enriched for histone modifications or proteins (*methods).* Genome structures: red, chromosome 1; blue, chromosome 2; green, chromosome 3. Epigenetic data: X axis, position on the chromosome (base pairs); y axis, relative enrichment of H3K9me2. (**B**) Chromosome coordinates for modifications were mapped onto the 3D genome structures and the relative density of the elements across the entire ensemble of structures was rotationally projected onto a plane. Heterochromatic loci enriched for the H3K9me2 were preferentially localized at the nuclear periphery in G1 synchronized *S. pombe* cells^9^. (**C**) Contour maps highlighting the top 15% of relative density signal are presented for G1 phase nuclear models restrained by connections captured in cells grown in defined media (EMM 2;^9^). The incorporation of biological restraints significantly altered the spatial distribution of heterochromatic loci. (**D**) The population level distribution of loci bound by Swi6 shows a preference for the nuclear periphery and about the SPB consistent with its known roles in RNAi and heterochromatin formation. (**E**) Actively transcribed euchromatin (H3K4me2) was centrally localized in G1 phase nuclear models.

**Figure 2:**
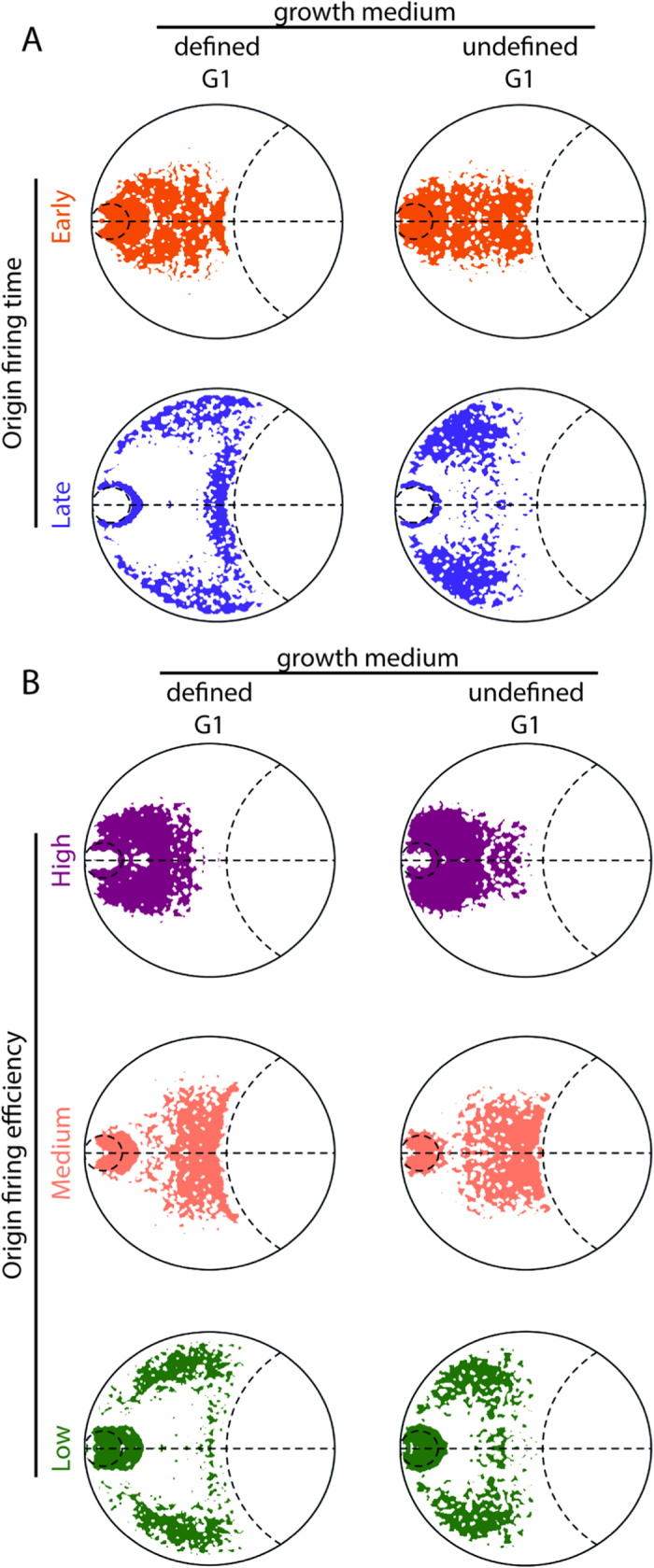
Replication origins are separated within the *S. pombe* nucleus. Contour maps of the locations of early and late firing replication origins^3^ in ensembles of contact-restrained genome structures that were generated as described in Fig. 1. (**A**) Early firing origins were central and late firing replication origins were located towards the periphery. Late firing origins were more centrally located in models derived from cells grown on undefined media^10^. (**B**) High efficiency origins fire in most cell cycles^3^ and are preferentially located toward the nuclear center and about the SPB. By contrast, low and medium efficiency origins^3^ are localized towards the nuclear periphery and nucleolar boundary, respectively. Low efficiency origins are more centrally located in cells grown in undefined media.

**Figure 3:**
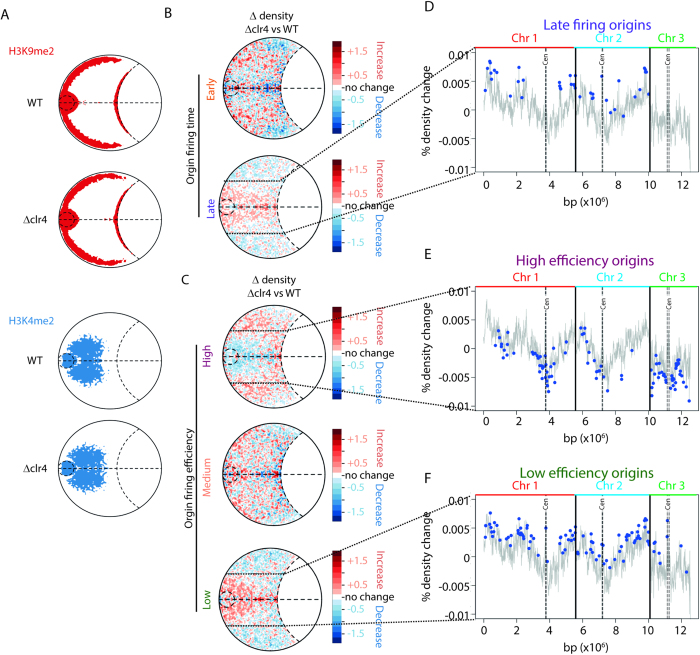
Deletion of the *S. pombe* histone methyltransferase (Clr4) altered the positioning of the origins of replication without affecting the global distribution of euchromatic and heterochromatic loci. (**A**) Contour maps revealed that loci that were decorated with euchromatin (H3K4me2) and heterochromatin (H3K9me2) marks in WT cells do not significantly shift their positions in *∆clr4* cells. (**B**) Relative density plots (% change per pixel) identified Clr4 dependent rearrangements in the positioning of early and late firing origins, which became more peripheral and central, respectively. (**C**) Similarly, deletion of C*lr4* resulted in the high and low efficiency origins moving towards the periphery and center of the nucleus, respectively. (**D**) Not all late firing origins move towards the nuclear center following deletion of Clr4. The relative contribution that each late firing origin made to the density change within the highlighted region was determined (methods). The grey line represents the % density change that each granule within the *S. pombe* genome model structures contributed to the highlighted region. Blue circles denote the origin positions under test. (**E**) High efficiency origins of replication on chromosome 3 and surrounding the centromeres of chromosomes 1 and 2 tended to move out from the nuclear interior following deletion of Clr4. (**F**) Low efficiency origins of replication that migrated into the nuclear interior tended to have more telomeric positions on the chromosomes. Relative contributions in (**E,F**) were determined as in D. Chr 1, Chromosome 1; Chr 2, chromosome 2; Chr3, chromosome 3; cen, centromere; bp x 10^6^, position within the concatenated *S. pombe* genome (*i.e.* Chr1,2,3).

**Figure 4:**
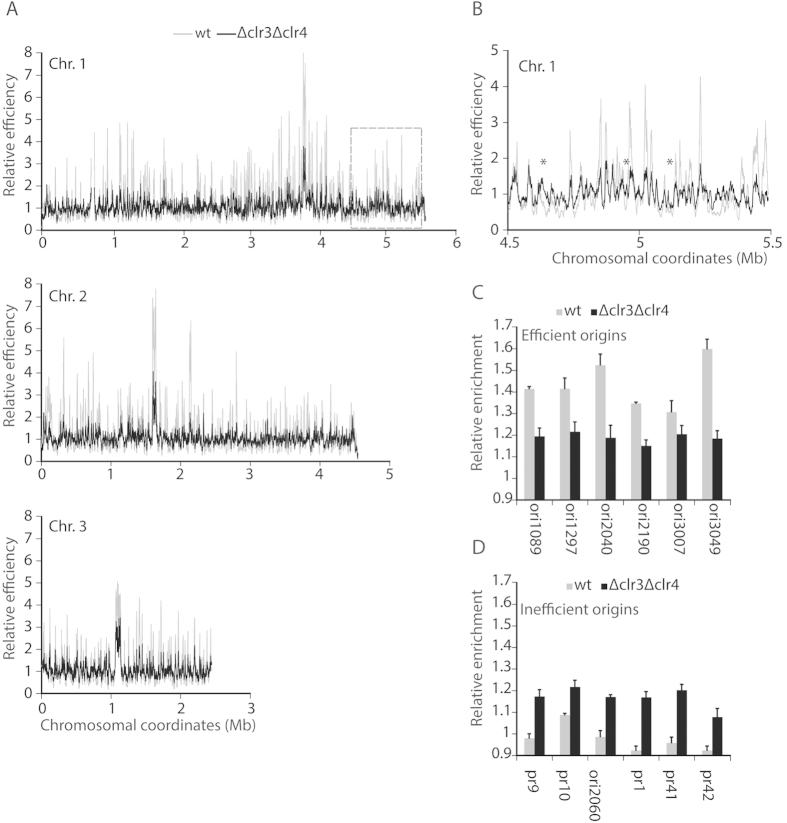
Genome wide reprogramming of replication origin usage in *S. pombe clr3∆clr4∆* cells. (**A**) Replicating DNA was labeled with BrdU in synchronous *cdc25-22* cultures, and analyzed by micro-array. Replication origins mapped along fission yeast chromosomes for wild type and *clr3∆clr4∆* cells and their relative efficiencies are shown (**B**) The replication profile of one efficient origin cluster on the right arm of chromosome I (boxed in panel (**A**) is shown at higher resolution. Asterisks indicate origins used more efficiently in *clr3∆clr4∆* cells than in wild type cells. (**C,D**) Quantitative real time PCR probing DNA content increase from G2 blocked cells to S-phase blocked cells at six efficient and six inefficient origins (Table S4). Two representative origins from each of the three *S. pombe* chromosomes are shown.

**Figure 5:**
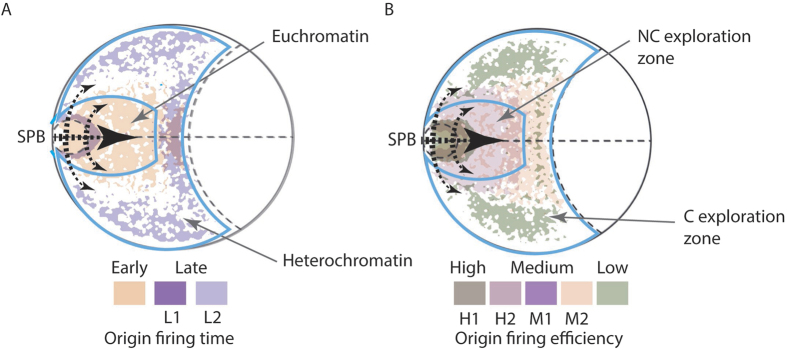
SPB-originating diffusion mechanism for replication origin firing. In this model limited concentration factors are activated within the spindle pole body associated area before diffusing through the nuclear chromatin to activate origins of replication. Chromatin architecture affects the diffusion rates and ability of diffusible factors to search for binding sites. Non-compact (NC) exploration within the euchromatin allows the exploration of large volumes, increasing the speed and efficiency of origin of replication activation. By contrast, compact (C) exploration retards the recognition and activation of origins of replication. Thus, chromatin architecture contributes to both: (**A**) timing of origin firing, and (**B**) the efficiency of origin firing. Black arrows denote the mobile factor(s) diffusion gradient. Diffusion through the centre of the nucleus is promoted by the structure of the euchromatin. L1, L2, H1, H2, M1 and M2 signify different origin environments that are defined by different degrees of overlap between early and late firing origins, high and medium efficiency, and medium and low efficiency origins, respecitvely.
