# Supplementary material for: A diffusion model for the coordination of DNA replication in Schizosaccharomyces pombe
Source: Sci Rep. 2016 Jan 5;6:18757. doi: 10.1038/srep18757 (PMC4700429; doi:10.1038/srep18757)
Supplement: Supplementary Information [file srep18757-s1.pdf]

Supplementary Materials for:

A diffusion model for the coordination of DNA replication in *Schizosaccharomyces pombe*.

Authors: T. Pichugina[1#], T. Sugawara[2#], A. Kaykov[3#], W. Schierding[1], K. Masuda[4], J. Uewaki[2], R.S. Grand[1†], J.R. Allison[5], R.A. Martienssen[6], P. Nurse[3,7], M. Ueno[2,4#], J.M. O'Sullivan[1,8*]

Supplementary Materials:

Figures S1-S9

Table S1-S4

Figures S1-S8

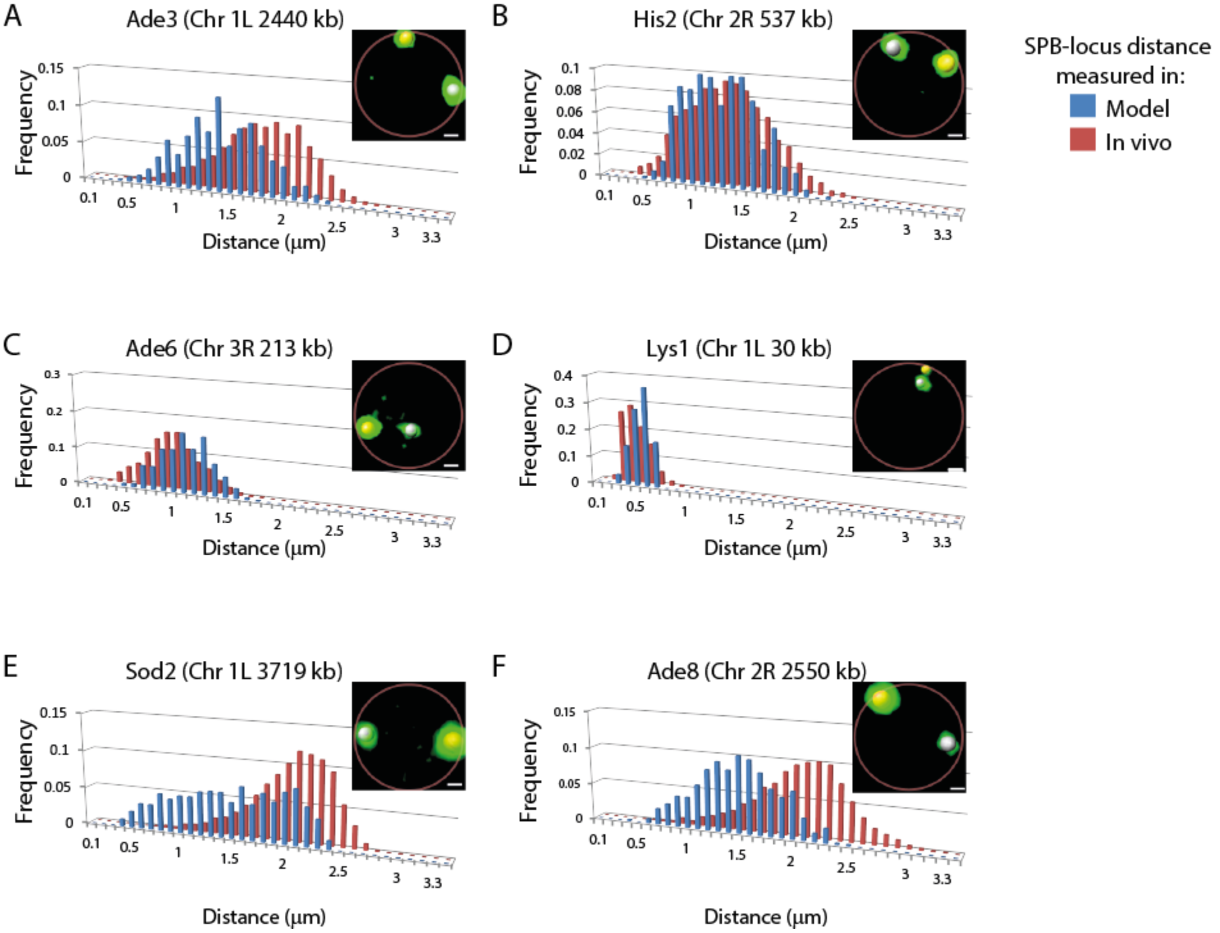

Figure S1. SPB-centromere distance distributions measured *in vivo* and calculated from the G1 phase genome structure models overlapped. Distances between the centers of the: A, *Ade3*; B, *His2*; C, *Ade6;* D, *Lys1*; E, *Sod2*; and F, *Ade8* genes and the SPB were measured microscopically *in vivo* (red) in unsynchronized cells and calculated from 500 *in silico* models of the *S. pombe* G1 phase genome organization (blue). Models incorporated connections captured from cells grown on EMM 2 medium (*1*). Representative live cell images are shown (inserts); white or yellow spot, focal points; red circle, nuclear periphery; scale bar = 0.5 (μm). The positioning of the *Ade8* locus reflected a more SPB proximal position for this locus in G1 phase cells. The distribution of the SPB-*Sod2* distances within the model ensemble was non-Gaussian consistent with the positioning of this sub-telomeric locus being affected by the fact that DNA cannot move past the nucleolar boundary in our models (*methods*). The linear coordinates used to map each locus onto the 3D structure are shown in brackets for each locus. Chr, chromosome. L, left arm; R, right arm.

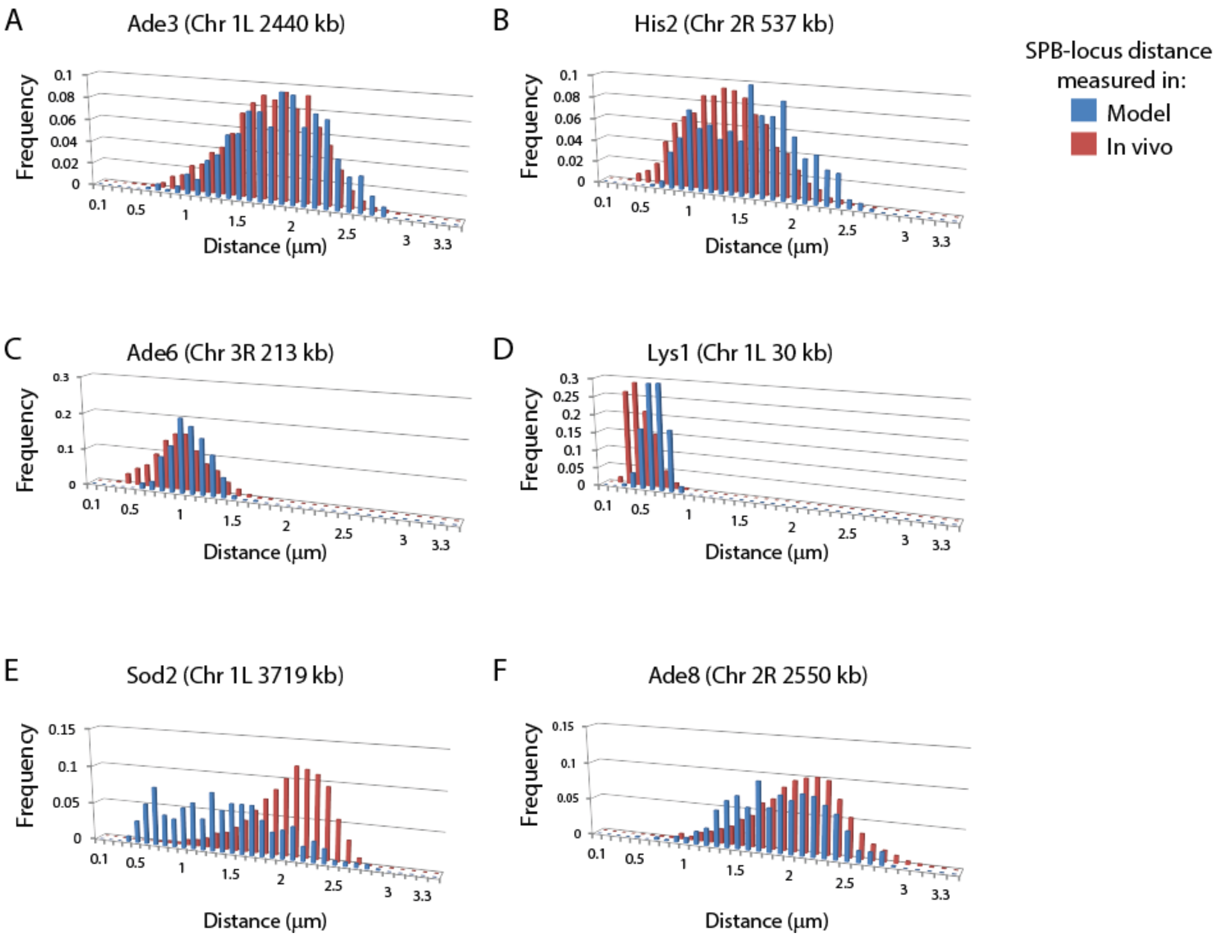

Figure S2. SPB-centromere distance distributions measured *in vivo* and calculated from the G2 phase genome structure models overlapped Distances between the centers of the: A, *Ade3*; B, *His2*; C, *Ade6;* D, *Lys1*; E, *Sod2*; and F, *Ade8* genes and the SPB were measured microscopically *in vivo* (red) in asynchronous cells and in 500 *in silico* models of *S. pombe* G2 phase genome organization (blue). Models incorporated connections captured from cells grown on EMM 2 medium (*1*). The distribution of the SPB-*Sod2* distances within the model ensemble was non-Gaussian and does not match the *in vivo* distribution. This is consistent with the positioning of this sub-telomeric locus being affected by the fact that DNA cannot move past the nucleolar boundary in our models (*methods*). The linear coordinates used to map each locus onto the 3D genome structures are shown in brackets for each locus. Chr, chromosome. L, left arm; R, right arm.

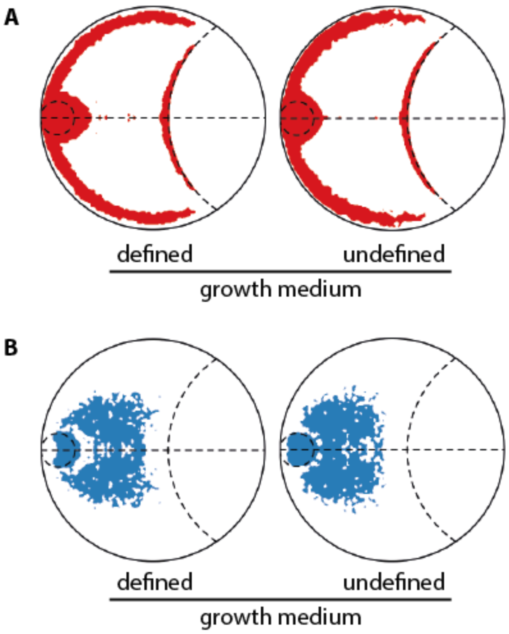

Figure S3 Growth medium had minor effects on the spatial positioning of the heterochromatic and euchromatic loci. Only minor differences were observed in the contour maps (top 15% of relative density signal) of **A** heterochromatic and **B** euchromatic loci in G1 phase contact-restrained nuclear models of cells grown in defined (EMM 2) and undefined ("rich") media.

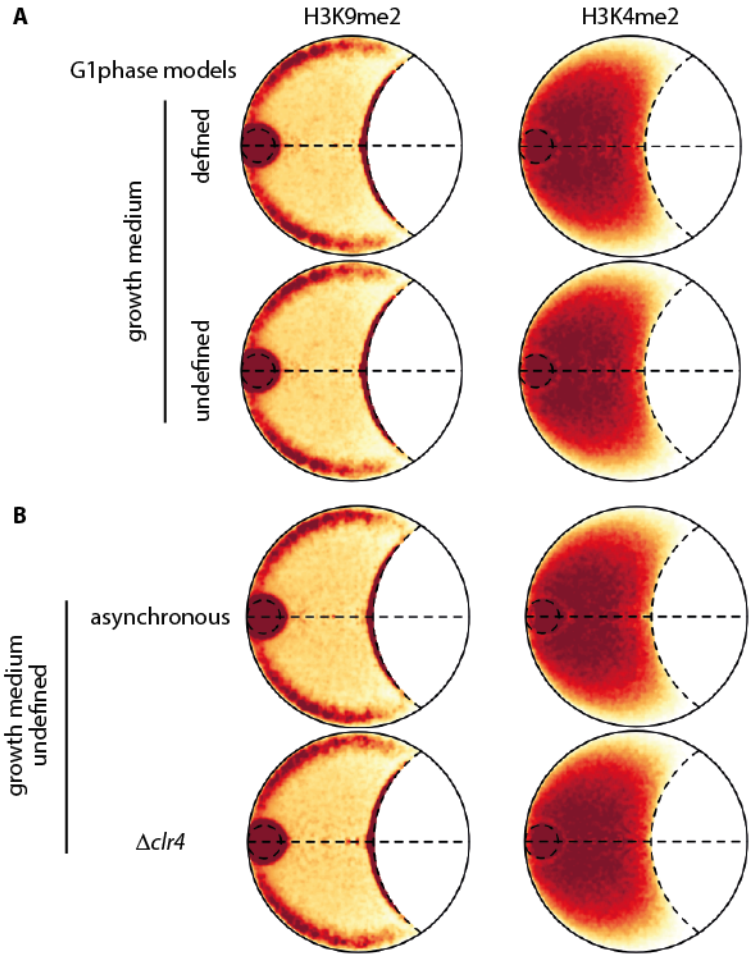

Figure S4 Overlap of the spatial organization of loci decorated with the H3K9me2 and H3K4me2 post-translational histone modifications in models of the *S. pombe* genome. A Linear coordinates for H3K9me2 and H3K4me2 modified loci were mapped onto the 3D polymer models as in Fig. 1 (methods). G1 phase models incorporated chromosomal contacts captured by proximity-ligation in temperature sensitive *cdc10* mutant *S. pombe* cells that were synchronized following growth in defined (EMM 2; (*1*)) or undefined (rich; (*7*)) medium. Loci marked with the H3K9me2 modification within the G1 phase nuclei models were predominantly peripheral but overlapped the H3K4me2 modified loci, particularly about the SPB. **B** Linear coordinates for H3K9me2 and H3K4me2 modified loci were mapped onto the 3D polymer models for asynchronous cells (*i.e. S. pombe* WT; (*7*)) and the *Δclr4* mutant (*S. pombe* SPK567; (*7*)), as in Fig. 1. As for the G1 phase models, loci marked with H3K9me2 and H3K4me2 modifications show a degree of overlap within the nuclear models.

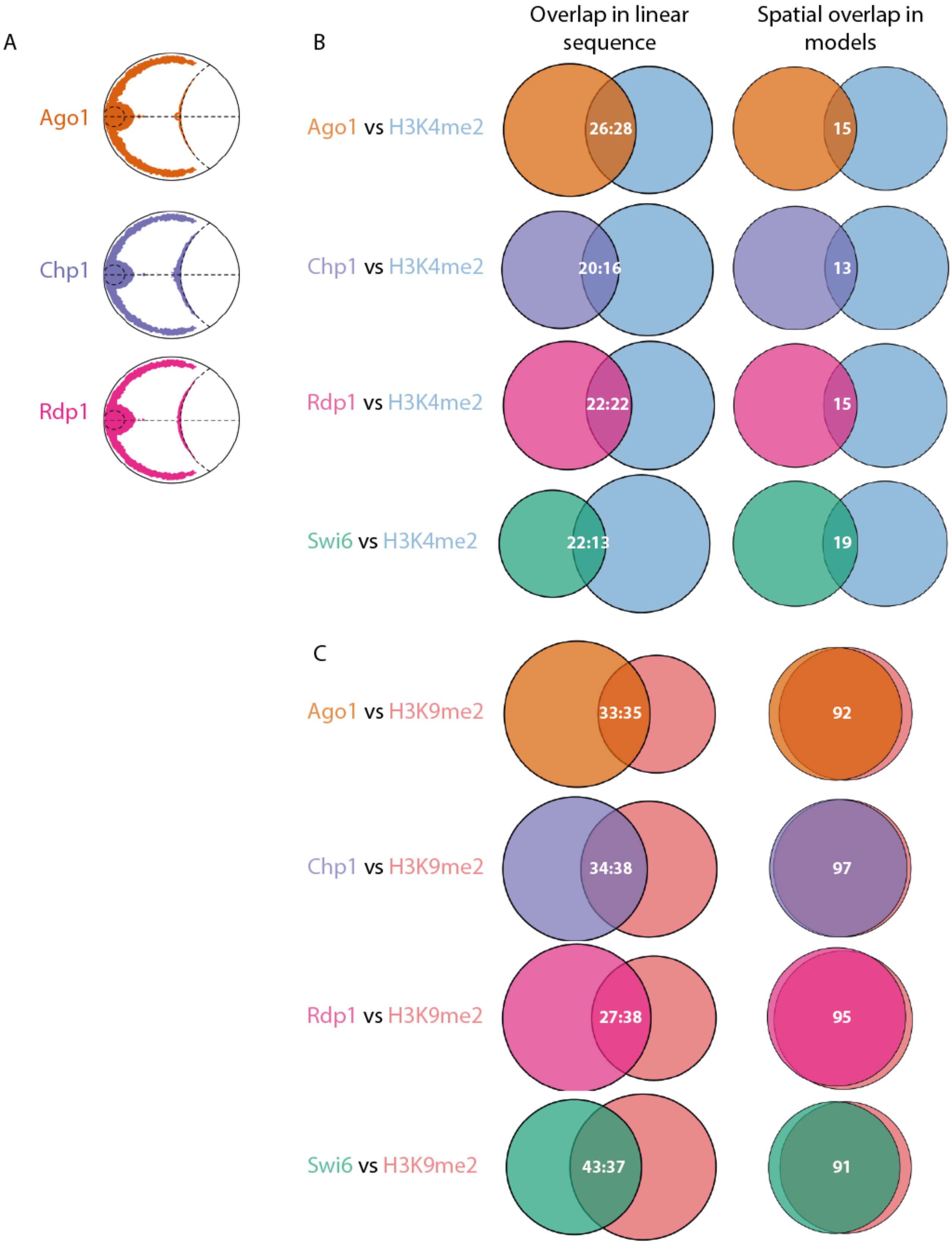

Figure S5. Binding sites for proteins involved in the establishment of heterochromatin overlap H3K9me2 modified regions in three-dimensions. A The population level distributions

of loci bound by Ago1, Chp1 and Rdp1 show preferences for the nuclear periphery and about the SPB. Percentage overlap of loci bound by the Ago1, Chp1, Rdp1 or Swi6 proteins, and loci that were modified with either **B** H3K4me2 or **C** H3K9me2 marks in the linear sequence and in the 2D rotational projection of the 3D interaction models were calculated and plotted as proportional Venn diagrams (*methods*). **B** There is little linear or spatial overlap between loci bound by the Ago1, Chp1, Rdp1 or Swi6 proteins and H3K4me2 modified loci. Numbers represent the overlap as a percentage of each set. **C** There is significant spatial overlap between loci bound by the Ago1, Chp1, Rdp1 or Swi6 proteins and H3K9me2 modified loci. These results are consistent with the segregation of the active and inactive chromatin within the nucleus and the known roles of the Ago1, Chp1, Rdp1 and Swi6 proteins (reviewed in (*17*, *18*)). Numbers represent the percentage overlap.

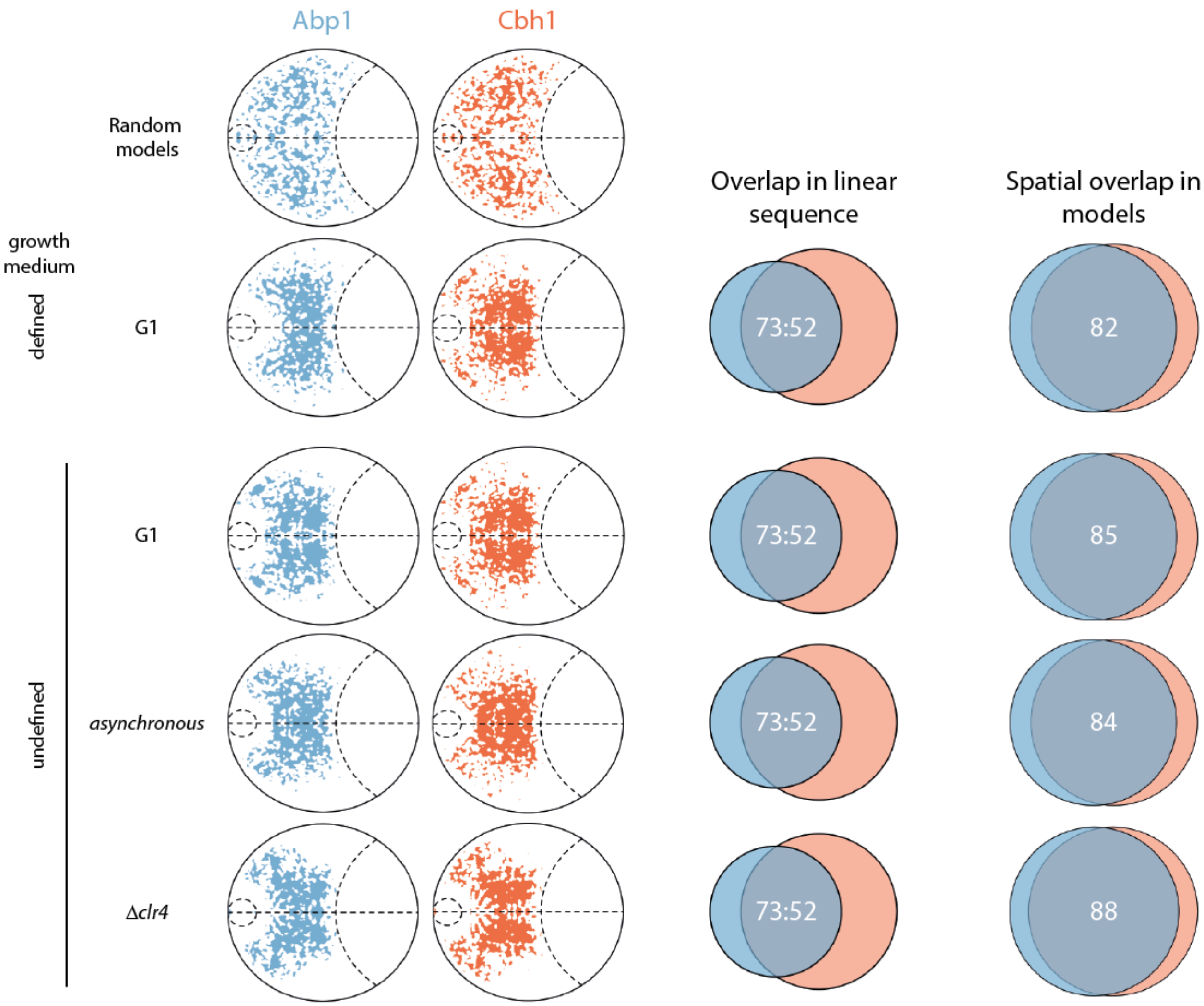

Figure S6. The linear and spatial profiles of binding sites for the Tf retrotransposon binding proteins Abp1 and Cbh1 are highly colocalized in all conditions. The relative density of loci bound by Abp1 and Cbh1 (*4*) was determined for the ensembles of contact-restrained models as described in Fig. 1. Percentage overlap in the linear and spatial models was calculated and plotted as proportional Venn diagrams (*methods*). The random models did not incorporate chromosomal contacts or other biological restraints. By contrast the other models incorporated biological restraints and chromosomal contacts that were captured by proximity-ligation in: G1, temperature sensitive *cdc10* mutant *S. pombe* cells that were synchronized following growth in defined (EMM 2; (*1*)) or undefined (rich; (*7*)) medium; asynchronous WT cells or *Δclr4* mutant (*S. pombe* SPK567) cells grown in undefined medium (*7*). Numbers represent the percentage overlap for each set. Loci bound by Abp1 and Cbh1 are centrally located in all contact-restrained models. However, the observed spatial overlap largely reflects the high level of overlap in linear positioning of these loci.

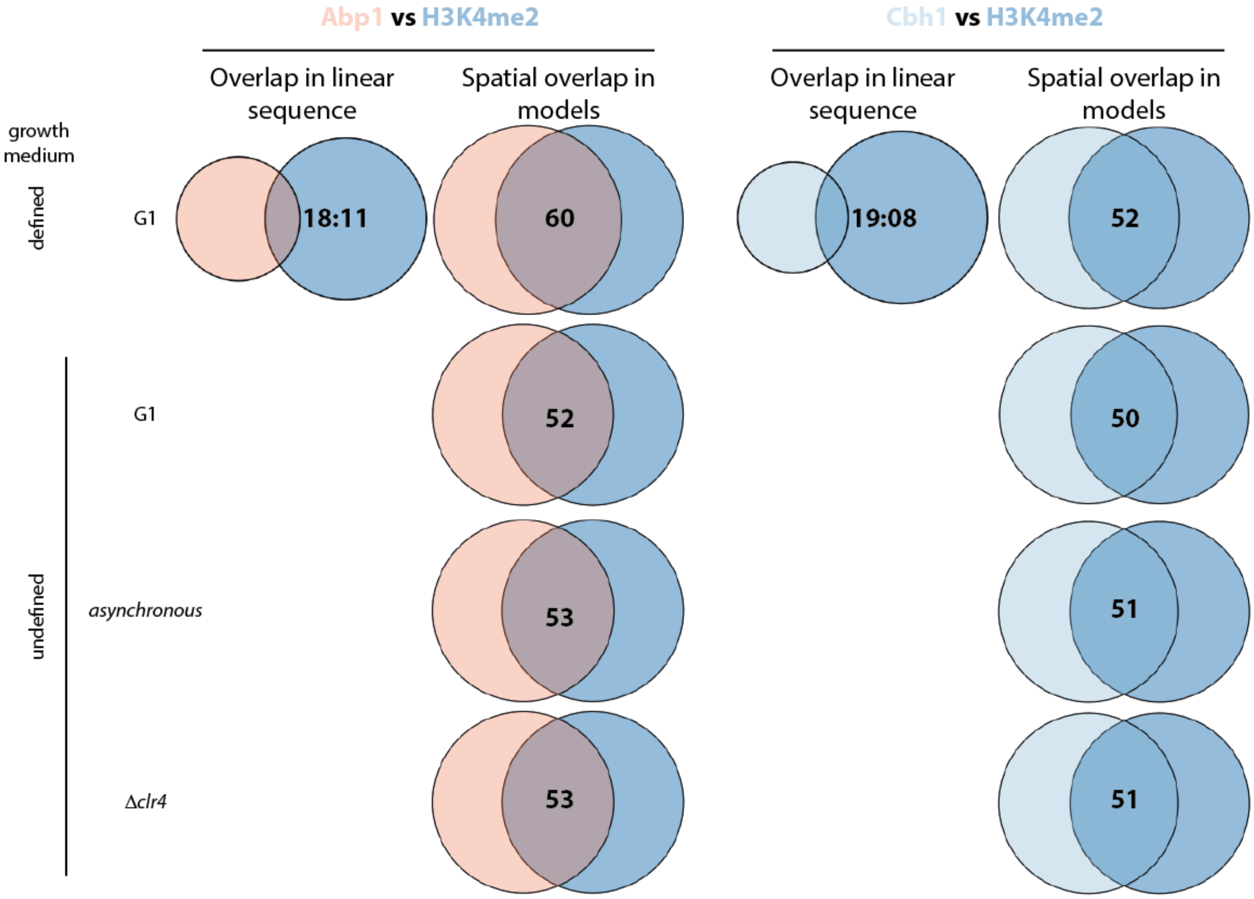

Figure S7. Abp1 and Cbh1 binding sites spatially overlap the H3K4me2 modification. The percentage overlaps in linear sequence or in relative spatial density of loci bound by Abp1 or Cbh1 and loci modified by H3K9me2 or H3K4me2 (*3*, *4*) were determined and plotted as Venn diagrams. Numbers represent the percentage overlap for each set. The linear overlap is represented by the percentage Abp1:percentage H3K4me2. The relative density of loci bound by Abp1 and Cbh1 (*4*) was determined for the ensembles of contact-restrained genome models as described in Fig. 1. Models incorporated chromosomal contacts that were captured by proximity-ligation in: G1, temperature sensitive *cdc10* mutant *S. pombe* cells that were synchronized following growth in defined (EMM 2; (*1*)) or undefined (rich; (*7*)) medium; asynchronous WT and *Δclr4* mutant (*S. pombe* SPK567) cells grown in undefined medium (*7*). The spatial colocalization of loci bound by Abp1 or Cbh1 and H3K4me2 loci is significantly greater than their linear clustering in all cases.

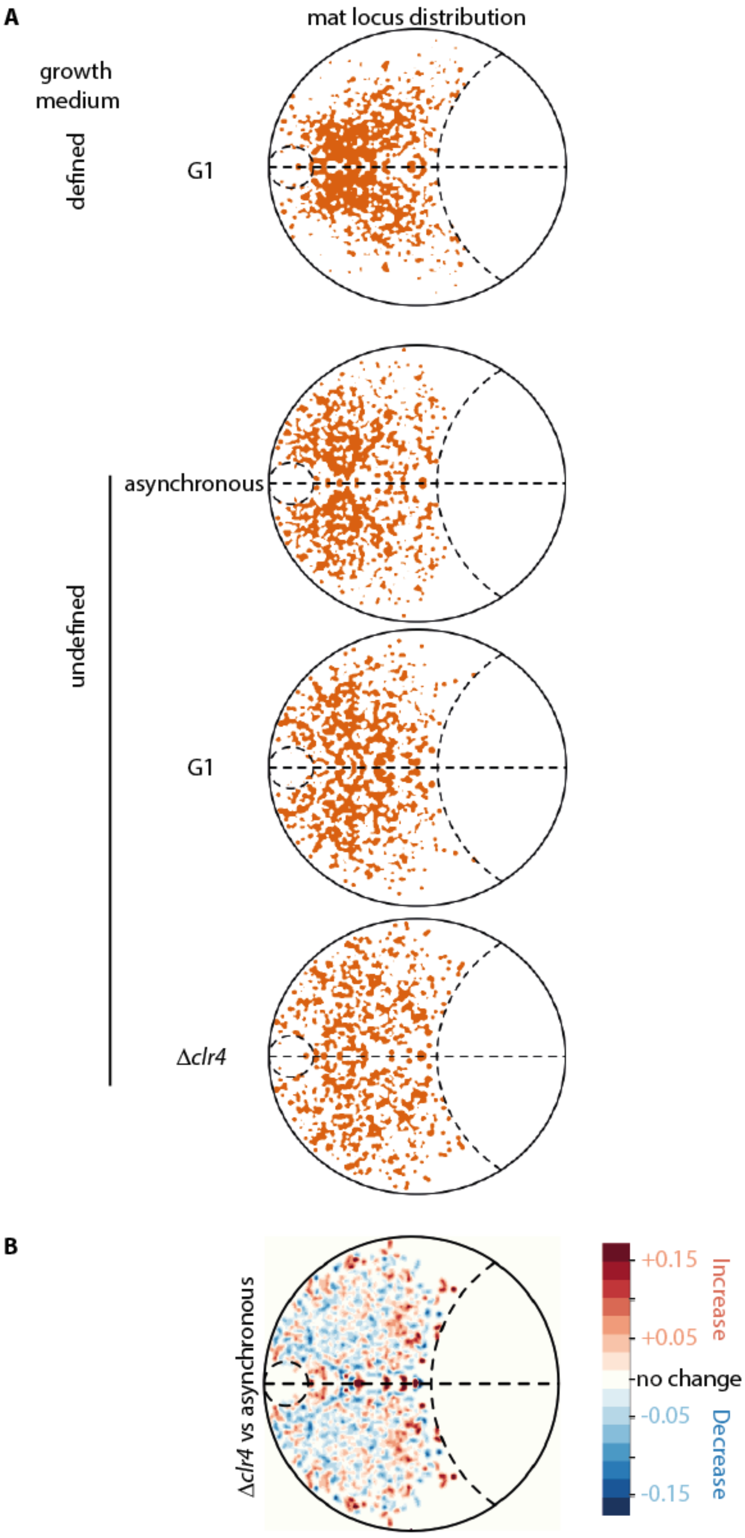

Figure S8. Deletion of the Clr4 methyltransferase correlates with a shift in the spatial distribution of the mating-type locus within the *S. pombe* nucleus. A The relative density of the mating-type (mat) locus (Chr2: 2129208-2137121) was determined for models that

incorporated chromosomal contacts that were captured by proximity ligation in: G1, temperature sensitive *cdc10* mutant *S. pombe* cells (synchronized following growth in defined (EMM 2; (*1*)) or undefined (rich; (*7*)) medium; and asynchronous WT and *Δclr4* mutant (*S. pombe* SPK567) cells grown in undefined medium (*7*). The mat locus distribution within models of the *S. pombe* genome organization is sensitive to the growth medium (compare G1 defined vs undefined) and the level of synchronization (compare asynchronous vs G1 grown in undefined media). **B** The spatial distribution of the mating-type locus (Chr2: 2129208-2137121) in models generated for the *Δclr4* and asynchronous (*S. pombe* wild-type) cells grown in rich medium (*7*) were compared to generate relative density plots (% change per pixel). Deletion of Clr4 resulted in the mating-type locus positioning becoming unrestricted.

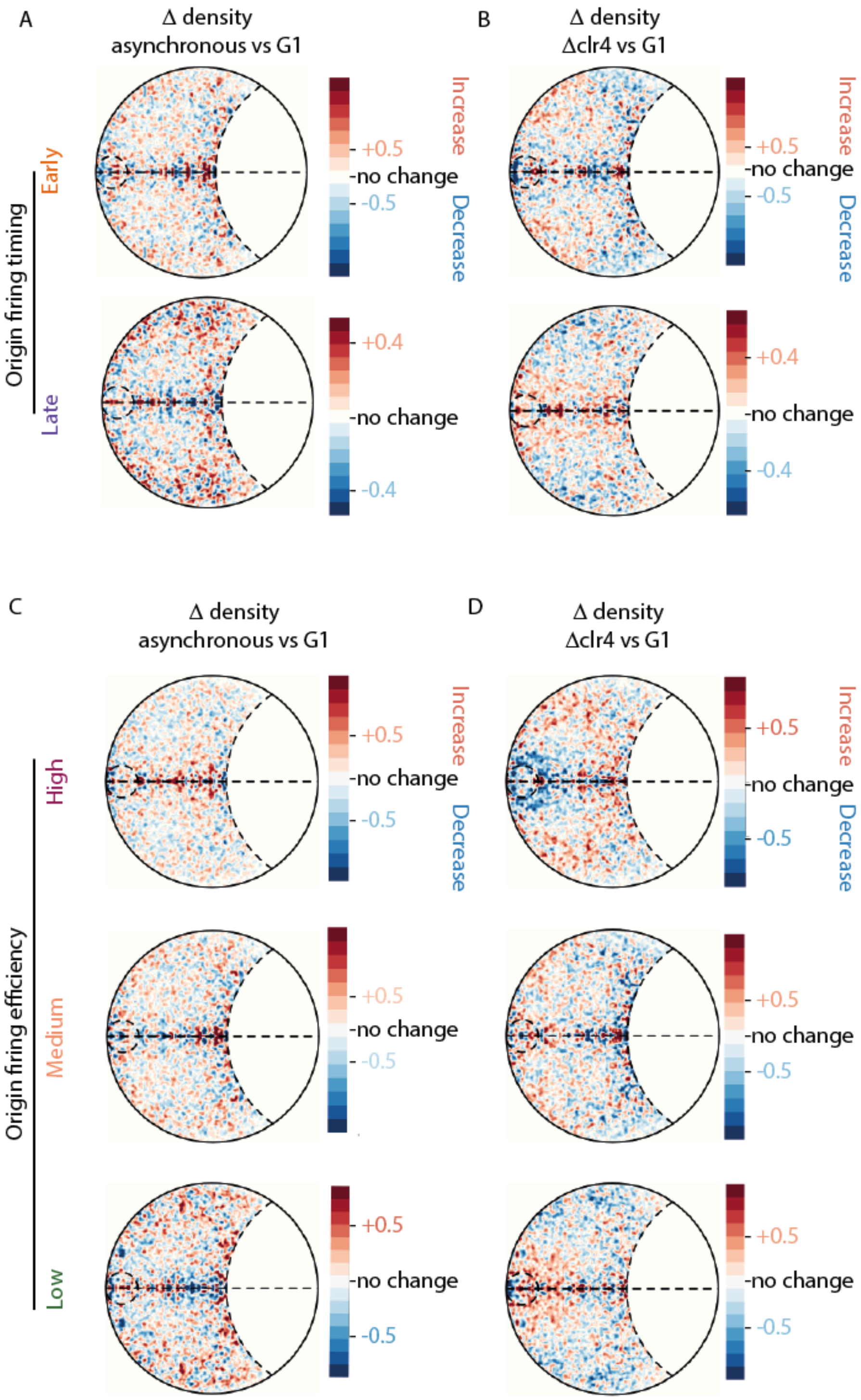

Figure S9. Deletion of the Clr4 methyltransferase correlates with a shift in the positions of the origins of replication that is not due to the loss of synchronization.

The relative density of early and late firing origins of replication (*5*) were prepared for models that incorporated chromosomal contacts captured by proximity ligation in: G1, temperature sensitive *cdc10* mutant *S. pombe* cells; asynchronous WT cells; and *Δclr4* mutant cells (*S. pombe* SPK567) that were grown in undefined medium (rich; (*7*)). The spatial distributions of the early

and late firing origins of replication were compared in models generated for **A** asynchronous and G1 phase nuclei and **B** *Δclr4* and G1 phase nuclei. The effects of deletion of the Clr4 methyltransferase are not explained by the loss of synchronization. The spatial distribution of the high, medium and low efficiency firing origins of replication were compared in models generated for **C** asynchronous and G1 phase nuclei and **D** *Δclr4* and G1 phase nuclei. As observed for the early and late firing origins of replication, the effects of deletion of the Clr4 methyltransferase are not explained by the loss of synchronization.

Table S1. *S. pombe* strains used in this study.

**strain**	Genotype	**Source**
KM023	*h*^{90} *ade6-M216 lys1-131 leu1-32 ura4-D18 sid4::sid4-GFP-HA-Kanr ade3[::kanr-ura4*$^{+}$*-lacOp] his7*$^{+}$*Pdis1-GFP-lacI-NLS*	This study
KM025	*h*$^{+}$ *ade6-M210 leu1-32 sid4::sid4-GFP-HA-Kanr lys1*$^{+}$*<<lacOp his7*$^{+}$*Pdis1-GFP-lacI-NLS*	This study
KM026	*h*^{90} *ade6-M216 leu1-32 lys1-131 ura4-D18 sid4::sid4-GFP-HA-Kanr his2[::kanr-ura4*$^{+}$*-lacOp] his7*$^{+}$*Pdis1-GFP-lacI-NLS*	This study
FY17781	*h*^{90} *ade6-M210 leu1-32 lys1-131 ura4-D18 sid4*$^{+}$*::GFP-kanr sod2.proximal[::kanr-ura4*$^{+}$*-lacO](l) his7*$^{+}$*::Pdis1-GFP-lacI-NLS*	NBRP
FY17780	*h*^{90} *ade6-M216 leu1-32 lys1-131 ura4-D18 sid4*$^{+}$*::GFP-kanr ade8[::kanr-ura4*$^{+}$*-lacO](j) his7*$^{+}$*::Pdis1-GFP-lacI-NLS*	NBRP
FY17791	*h*$^{-}$ *leu1-32 lys1-131 ura4-D18 sid4*$^{+}$*::GFP-kanr ade6[::kanr-ura4*$^{+}$*-lacO](j) his7*$^{+}$*::Pdis1-GFP-lacI-NLS*	NBRP
FY15592	*h*^{90} *ade6-216 leu1-32 lys1-131 ura4-D18 ade3[::kanr-ura4*$^{+}$*-lacOp] his7*$^{+}$*::lacI-GFP*	NBRP
FY15591	*h*^{90} *leu1-32 lys1 ura4-D18 ade6-M216 his2[::kanr-ura4-lacOp] his7*$^{+}$*::lacI-GFP*	NBRP
FY14816	*h*^{90} *ade6-216 leu1-32 lys1-131 ura4-D18 sid4::sid4-GFP-HA-Kanr*	NBRP
FY15550	*h*$^{+}$ *ade6-M210 leu1-32 ura4-D18 his7*$^{+}$*::lacI-GFP lys1*$^{+}$*::lacOp*	NBRP
PN10499	*h-(Msmt-0) leu1-32::pFS181[Padh1-hENT1 leu1+] his7-336 pJL218[Padh1-hsvTK his7+] cdc25-22*	Kaykov & Nurse (2015)
PN10607	*h-(Msmt-0) leu1-32::pFS181[Padh1-hENT1 leu1+] his7-336 pJL218[Padh1-hsvTK his7+] clr3Δ::KanR clr4Δ::LEU2 cdc25-22*	Kaykov & Nurse (2015)

Table S2. Late-firing origins that were responsible for the top 5% of the observed changed in density at the nuclear center in the Δ*clr4* models.

	late firing origins	
Chromosome	Position (kbp)	Δ density (%)
1	231	0.007
1	367.5	0.008
1	399	0.008
1	441	0.006
1	476	0.005
1	651	0.007
1	798	0.007
1	2100	0.005
1	2142	0.007
1	2404.5	0.005
1	4599	0.005
1	5250	0.006
1	5316.5	0.004
1	5565	0.006
2	1592.5	0.006
2	2628.5	0.005
2	4182.5	0.007
2	4231.5	0.007
2	4238.5	0.007
2	4298	0.007

Table S3. Low efficiency origins that were responsible for the top 5% of the observed changed in density at the nuclear center in the Δ*clr4* models.

Low efficiency origins		
Chromosome	Position (kbp)	Δ density (%)
1	115.5	0.005
1	126	0.005
1	360.5	0.006
1	399	0.008
1	441	0.005
1	651	0.006
1	798	0.005
1	2086	0.005
1	2142	0.007
1	2243.5	0.005
1	2621.5	0.005
1	2642.5	0.005
1	3773	0.005
1	5250	0.005
1	5565	0.005
2	3829	0.005
2	4182.5	0.006
2	4203.5	0.005
2	4231.5	0.007
2	4298	0.006
3	1060.5	0.006

Table S4. Primers used for quantitative real-time PCR. The chromosomal coordinates of all amplified loci are shown.

**PCR product**	**Amplified Locus** **(5' position on plus strand)**	Forward primer	Reverse primer
ori1089	Chr. I - 1192128	TGCTCCCAATCATCCGACACAC	AATCCCAAAACTGTGCAAACTGCA
ori1297	Chr. I - 4090478	TAGCGCTTTTGATAGGTGTCGTGG	AGGTGGACCGAAATTATCCCTTCC
ori2040	Chr. II - 1261698	GCTTCCATAGACAGCGCCATTCTA	CCTCAGCTTCAGCAGGTAGCAAAG
ori2190	Chr. II - 2788497	GCTTACATCCAGAGGATGCGAACA	GTGGAGGTACACAGTGATCGGGATT
ori3007	Chr. III - 123351	TATGTATTTGGCGCTAAACAATCTCT	TACAATGACAAGATAATATTTATAGCGA AAATTT
ori3049	Chr. III - 1412755	CCCGTGAAATCCAGACTGCTGTT	CGGATTTGGTACCTTCGGTAACG
pr9	Chr. I - 3453751	GGAATATGCGATGAGTTCGCTTGA	CGAATGGACTTTTTATCGCGCAC
pr10	Chr. I - 3490195	TGGGAAAATGGCACCTTTACTACAA AG	AATAGCTGTTGTCGTTTTTGAAGGTTGA T
ori2060	Chr. II - 1867928	TTCAGGGCTCAAAGTTAGAAAAATC AAGT	CCCGAAATTGCACGGATAGTATAATT
pr12	Chr. II - 730804	CACATCCCTTTTGCCAAACAGCTA	GGTAAACACGATGTCGACGGTCC
pr41	Chr. III - 1212350	ATTTGGTCCATTGGAGCACAGAAAC	CTGCCATGCTAGGTTCCATCACAC
pr42	Chr. III - 1383748	CATGCTCTCGAAAACTATGGATGGG	CGATATCATGCACGCCATAAACGA
pr_reference	Chr. II - 1843580	GCACAGCAAAATGCTAGAGCCAAA	CAACTCGTTTAGGAATTGCAGATGAAT
